# Improved Hybrid Percolation–Ultrasound Extraction of Bioactive Compounds and Their Application as Nettle and Sage-Derived Biostimulants in Tomato and Pepper Crops

**DOI:** 10.3390/foods14223900

**Published:** 2025-11-14

**Authors:** Ana-Maria Tăbărașu, Nicolae-Valentin Vlăduț, Florin Nenciu, Petru Cârdei, Iuliana Găgeanu, Luminița Catană, Mihaela Begea, Mihai-Gabriel Matache, Dragoș-Nicolae Anghelache, Ioan-Cătălin Persu, Teofil-Alin Oncescu

**Affiliations:** 1 International Projects and Relations Department, National Institute of Research-Development for Machines and Installations Designed to Agriculture and Food Industry-INMA, 6 Ion Ionescu de la Brad Avenue, 013813 Bucharest, Romania; anamariatabarasu22@yahoo.com (A.-M.T.); iulia.gageanu@gmail.com (I.G.); alin.oncescu93@gmail.com (T.-A.O.); 2 Faculty of Biotechnical Systems Engineering, National University of Sciences and Technology Politehnica Bucharest, 060042 Bucharest, Romania; 3 Testing Department, National Institute of Research-Development for Machines and Installations Designed to Agriculture and Food Industry-INMA, 6 Ion Ionescu de la Brad Avenue, 013813 Bucharest, Romania; valentin_vladut@yahoo.com (N.-V.V.); petru_cardei@yahoo.com (P.C.); gabimatache@yahoo.com (M.-G.M.); dragos1989anghelache@gmail.com (D.-N.A.); persucatalin@yahoo.com (I.-C.P.); 4 Technology and Business Incubator INMA-ITA, National Institute of Research-Development for Machines and Installations Designed to Agriculture and Food Industry-INMA, 013811 Bucharest, Romania; 5 National Research & Development Institute for Food Bioresources (IBA Bucharest), 020323 Bucharest, Romania; lumi_catana@yahoo.co.uk

**Keywords:** statistical analysis, hybrid percolation–ultrasound extraction, greenhouse, polyphenols

## Abstract

The present research aimed to improve the extraction efficiency of polyphenolic compounds from nettle and sage, using an improved hybrid ultrasound—percolation extraction method. A factorial experimental design was employed to systematically evaluate the influence of key extraction parameters: pressure (5, 6, and 7 bar), extraction time (60, 90, and 120 min), and ultrasound power (80, 100, and 120 W) on the total polyphenol content (TPC) of the resulting extracts. The obtained extracts were comprehensively analyzed in terms of total polyphenol concentration, micro- and macronutrient content, and antioxidant activity. Based on the results, optimal extraction conditions were determined and subsequently used to formulate a biostimulant solution derived from nettle and sage. To validate the agronomic efficacy of the formulated biostimulant, greenhouse trials were conducted on tomato (*Solanum lycopersicum*) and pepper (*Capsicum annuum*) plants. The impact of the treatment was assessed by monitoring vegetative growth (total dry mass), chlorophyll content, and crop productivity. The central hypothesis is that systematic variation of the hybrid ultrasound-assisted percolation process parameters will reveal a range capable of significantly improving the polyphenol yield and antioxidant activity of nettle and sage extracts. We further used these improved extracts to increase certain health indicators and overall productivity in tomato and pepper plants. At the end of the growing period, the treated plants showed an increase in dry mass of 22% for tomatoes and 20% for peppers relative to controls samples. Furthermore, productivity showed a substantial increase, rising by 38.6% for tomatoes and 53% for peppers. Chlorophyll content also increased by up to 20% in tomatoes and up to 22% in peppers, showing better plant health.

## 1. Introduction

Polyphenols are natural bioactive compounds synthesized by plants, characterized by aromatic structures with one or more hydroxyl groups, representing the largest class of secondary metabolites in the plant kingdom [1,2]. Classified into flavonoids, phenolic acids, stilbenes, and lignans, these compounds play an essential role in plant defense against oxidative stress, UV radiation, pathogens, and abiotic stress [3,4]. Due to several extracts properties (antioxidant activity, cellular stabilization, osmotic regulation, and the ability to neutralize heavy metals), polyphenols significantly contribute to plant adaptation to unfavorable environmental conditions, thereby supporting sustainable agriculture [5,6,7,8].

In the current transition toward ecological agricultural practices, extracts obtained from medicinal and aromatic plants are attracting increasing interest due to their rich composition of some bioactive compounds, particularly polyphenols. These natural secondary metabolites present antifungal, antioxidant, and insecticidal properties, which offer an essential role in crop protection and in stimulating plant physiological processes [9]. Foliar application of polyphenolic plant extracts has proven effective in increasing the content of photosynthetic pigments, stimulating vegetative development, and enhancing agricultural yields [10]. At the same time, these natural solutions reduce the pressure exerted by pathogens and pests, contributing to strengthening plant resistance against biotic and abiotic stress factors [11]. By combining stimulatory physiological effects with phytosanitary protection, polyphenolic plant extracts represent a viable and sustainable alternative to the use of conventional agrochemicals, promoting integrated agriculture that is safer for both the environment and human health [12,13].

### 1.1. Nettle and Sage Plants Characteristics and Potential Use in Agriculture

Nettle (*Urtica dioica*) is a perennial herbaceous plant from the *Urticaceae* family, distinguished by its ecological adaptability and high content of bioactive compounds, among which polyphenols occupy a central place, being involved in antioxidant, anti-inflammatory, and antimicrobial mechanisms [14]. The phytochemical profile of nettle is complex and influenced by factors such as the geographical region, the plant part used, and the applied extraction method [15]. In this context, modern extraction methods prove increasingly advantageous due to shorter processing times, the use of moderate temperatures, and the possibility of precise control of operational parameters, which allows the preservation of sensitive bioactive compounds [16,17]. Recent studies [15] report the presence of phenolic acids of biological importance, such as gallic acid, ferulic acid, and chlorogenic acid, along with flavonoids such as kaempferol and luteolin. Furthermore, study [18] complements this view by revealing that fresh nettle leaves contain the highest polyphenol content, particularly rutin, quercetin, kaempferol, ferulic acid, and p-coumaric acid.

Another plant that contains bioactive compounds with high biological activity is sage (*Salvia officinalis* L.), recognized particularly for its high content of phenolic compounds, which contribute to its antioxidant, anti-inflammatory, and antimicrobial activities.

Polyphenols from *Salvia officinalis* leaves can be extracted by means of high hydrostatic pressure (HHP) at 600 MPa, 60 °C, for 5 min, using 30% ethanol as solvent [19]. Although efficient (the total polyphenol content reached 3811.84 mg RAE (Rutin Acid Equivalents)/100 g), this method involves harsh conditions, unlike mild extractions at room temperature, which are more suitable for heat-sensitive compounds. Among the polyphenols identified were rosmarinic acid, chlorogenic acid, catechin, luteolin, and apigenin. In study [20], sage was evaluated for its phytochemical composition and biological activity, using an extract obtained by maceration with 70% ethanol for 30 min, with the main compounds identified being rutin, catechin, chlorogenic acid, carvacrol, and naringin.

Due to their high content of bioactive compounds, nettle and sage extracts have demonstrated effectiveness in plant protection and yield improvement, while also representing a viable ecological alternative for treatments applied to greenhouse-grown vegetables, especially in the context of sustainable agriculture.

### 1.2. Polyphenols Main Extraction Technologies

Conventional extraction methods such as maceration or Soxhlet extraction are still employed, although they involve long processing times and, in some cases, high temperatures that may affect compound stability. For example, in study [21], polyphenols were extracted using two classical methods, maceration (24 h at room temperature) and Soxhlet extraction (6 h), with ethanol and hexane as solvents. The most efficient combination was Soxhlet extraction with ethanol, which yielded 13.35 mg GAE (Gallic Acid Equivalents)/g DW (Dry Weight), whereas maceration with hexane resulted in the lowest yield (6.12 mg GAE/g DW), highlighting the importance of both extraction method and solvent in efficiency. In study [22], polyphenols were extracted from nettle leaves and roots by means of cold maceration, using water as the solvent, at 4 °C for 72 h. The total polyphenol content was 15.30 mg GAE/g for leaves and 1.65 mg GAE/g for roots. Among the phenolic compounds identified were rutin, isoquercetin, and 2-O-caffeoyl malic acid.

According to study [23], modern extraction techniques such as microwave-assisted extraction (MAE) and ultrasound-assisted extraction (UAE) have proven to be more efficient and milder compared with conventional methods, allowing the recovery of significant amounts of polyphenols in a short time of only 30 min. The polyphenol content obtained was 156.07 mg/L by MAE and 71.44 mg/L by UAE, compared with 295.99 mg/L via Soxhlet extraction, but at longer times and higher temperatures. Among the identified compounds were caffeic acid, 5-O-caffeoylquinic acid, rutin, and quercetin. In an ecological approach [24], ultrasound-assisted extraction combined with deep eutectic solvents (UAE-DES) was applied, yielding high concentrations of polyphenols and strong antioxidant activity. The high efficiency was associated with the presence of compounds such as caffeoyl malic acid, 5-O-caffeoylquinic acid, caffeic acid, and quercetin 3-O-rutinoside.

### 1.3. Application of Medicinal Plant Extracts for Vegetable Treatment in Greenhouses

There are several specific growing difficulties encountered by vegetables cultivated in greenhouses that can be reduced using aqueous extracts obtained from medicinal plants [25,26]. Studies showed that treatments applied at different developmental stages may lead to significant increases in plant height and fresh biomass (up to 68%), as well as stimulation of phenolic compounds and enzymatic and non-enzymatic antioxidants. Plant extracts led to an increase in biomass, photosynthetic pigments, polyphenol content, and ascorbic acid, confirming their usefulness in optimizing production in protected systems.

Furthermore, several studies [27,28] provide a comprehensive synthesis of the potential of aromatic plant extracts in controlling soil-borne pathogens, due to their antifungal, antibacterial, and nematicidal activity. Nettle and sage extracts have demonstrated effectiveness in controlling agricultural pests, reducing infestations with aphids, mites, and nematodes, and limiting the transmission of viral diseases [13,29,30,31]. These effects are associated with the bioactive compounds present in the plants, whose concentrations can be enhanced through modern extraction methods. Compared with maceration or extraction by boiling at high temperatures, ultrasound-assisted extraction allows for higher yields of polyphenols to be obtained in a shorter time and at moderate temperatures [32,33,34]. In particular, UAE has generated high contents of phenolic compounds from both sage [33] and nettle [23], especially when solvents such as 50% ethanol or water were used [35].

The study addresses several limitations related to conventional methods for polyphenolic compound extraction, which often yield suboptimal results and lack systematic evaluation of extraction parameters. The objective of this study was to improve the extraction process of polyphenolic compounds from nettle and sage through the application of an innovative hybrid technology, namely, percolation assisted by ultrasound. The research involved a factorial analysis (27 experiments per plant, with 3 repetitions for each sample) of the influence of the main technological parameters: pressure (5, 6, and 7 bar), extraction time (60, 90, and 120 min), and ultrasound power (80, 100, and 120 W) on the total polyphenol content. The originality of this work lies in combining this hybrid extraction method with a detailed statistical evaluation of the experimental results, within a scientific context where percolation assisted by ultrasound is insufficiently explored in the literature. The hypothesis of this part of the study is that optimizing pressure, extraction time, and ultrasound power in the hybrid ultrasound percolation process will lead to a significant increase in total polyphenol content.

Based on the obtained values for total polyphenol content, the optimal extraction conditions were selected, and the resulting extracts based on nettle and sage were used to formulate a biostimulant solution. This solution was applied to tomato and pepper plants cultivated in greenhouses, while the chlorophyll content, total plant dry mass, and productivity were evaluated as the main indicators.

The present study offers important practical value for producing superior extracts for vegetable protection and fertilization. Plant-based biostimulants can enhance crop yield, improve plant physiological status, and reduce reliance on chemical fertilizers, supporting more sustainable greenhouse practices. Although the benefits of extracts for treating various vegetable crops are consistently reported in the literature [36,37], the efficiency of the ultrasound-assisted percolation process has not been adequately addressed.

## 2. Materials and Methods

The research focuses on improving a hybrid percolation–ultrasound extraction method to increase the efficiency of bioactive compound recovery from sage and nettle. The aim was to maximize the extraction yield of metabolites having bioprotective and fertilizing functions, thereby enhancing their applicability in sustainable agriculture. Although ultrasound and percolation-based extraction techniques have been widely studied under controlled laboratory conditions, a critical gap persists regarding their validation in real cultivation environments. To address this gap, the present work offers a preliminary experimental validation of the obtained extracts in commercial greenhouse systems, using pepper and tomato crops as test models.

### 2.1. Experimental Design

**Figure 1:**
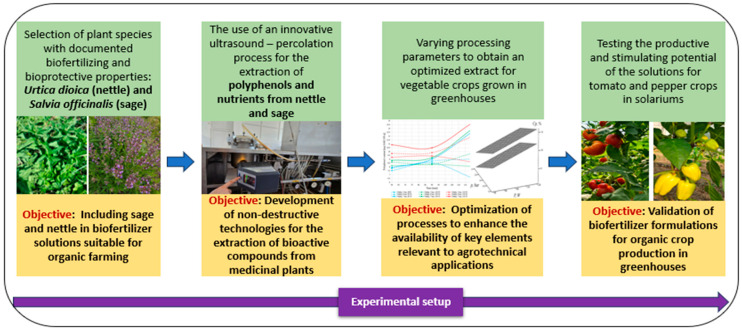
Experimental workflow for the extraction and validation of biostimulants from *Urtica dioica* and *Salvia officinalis*.

Figure 1 illustrates a sequential approach for extracting and validating plant-based biostimulant intended for organic greenhouse systems.

This approach capitalizes on renewable plant resources, such as *Urtica dioica* and *Salvia officinalis*, to support sustainable agricultural practices. Extracts from these plants, obtained through a mild ultrasound-assisted percolation method, preserve essential bioactive compounds beneficial for the development of agricultural crops.

The process was optimized to maximize nutrient availability and was tested on greenhouse crops to validate its practical applicability. The method is environmentally friendly, free of chemical inputs, and compliant with European standards for organic farming. By using these extracts, soil health and plant resilience are improved, contributing to reduced dependence on synthetic inputs. Moreover, the method is versatile and can be adapted to a wide range of crops. Overall, this solution provides a concrete example of integrating technological innovation into sustainable agriculture.

The selection of design parameters for the hybrid percolation process was based on preliminary screening tests and the operational constraints of the equipment used. The maximum ultrasound power was set at 120 W, as higher values (for example, 140 W) resulted in a lower Total Polyphenol Content (TPC) compared to that obtained at 120 W. The extraction pressure was limited to 7 bar due to the technical specifications of the percolation system. The maximum extraction time of 120 min was chosen to ensure the efficiency of the hybrid extraction process, allowing high recovery within a short time frame and thus distinguishing this method from conventional techniques.

### 2.2. Collection of Plant Material

**Figure 2:**
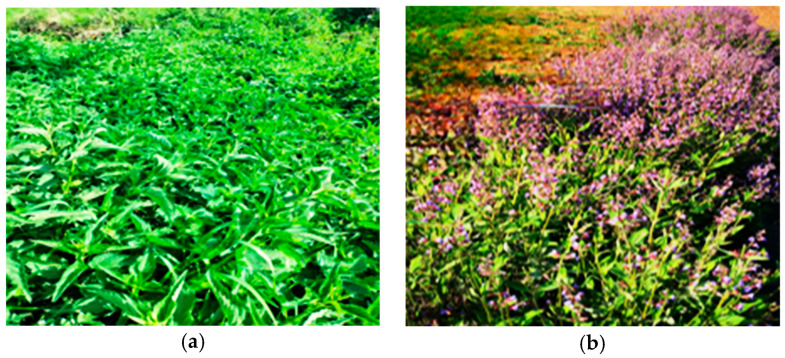
Organic experimental crops of nettle (**a**) and sage (**b**), used as raw material for extraction.

The plant raw material used in this study consisted of nettle and sage (Figure 2), harvested under controlled conditions to ensure the quality of the phytomaterial. Nettle plants, with an average height of 30–35 cm, were collected manually from spontaneous flora at approximately 5 cm above soil level. Sage plants, with a height of about 40–45 cm, were harvested mechanically at 10–15 cm above soil level from an experimental crop established in 2022 in Bucharest, a region with a temperate-continental climate.

### 2.3. Preparation of Plant Material

Drying of the plant material was performed naturally at room temperature, on specially designed sieves that allowed good air circulation and prevented the occurrence of mold or the degradation of heat-sensitive phytocompounds. Prior to cutting, moisture content was determined using a Shimadzu MOC63u (Shimadzu, Kyoto, Japan) moisture analyzer. Nettle showed a moisture content of 10.19%, and sage 10.29%, values indicating proper drying and optimal preparation for extraction [38,39].

After moisture determination, the plants were uniformly cut using the HerbCut Timatic equipment (Tecnolab srl, Spello, Italy), designed for the processing of medicinal and aromatic plants. Cutting was performed to a fragment size of approximately 3 cm to ensure homogeneity of the extraction process.

**Figure 3:**
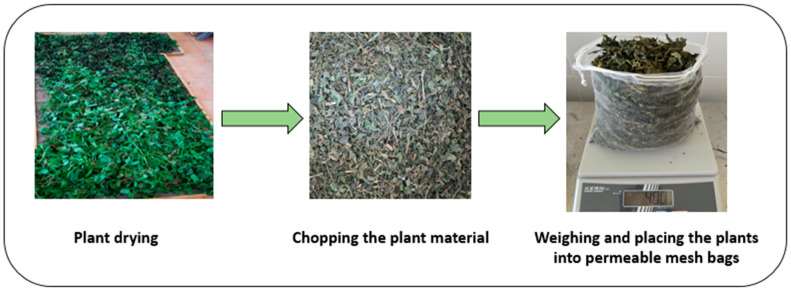
Schematic representation of plant material preparation before extraction.

For the experimental treatments, 400 g of dried and cut material from each plant species was weighed and placed into special permeable bags, designed to allow uniform contact with the solvent during the extraction process (Figure 3).

### 2.4. Description of the Hybrid Pressure Percolation–Ultrasound Extraction Equipment and Extraction Parameters

For percolation extraction, a TIMATIC Duo device (Tecnolab srl, Spello, Italy) was used, originally equipped with two extraction chambers (24 L and 12 L, respectively). However, for the purpose of this experiment, the equipment was modified: one chamber was removed, and only the 12 L chamber was retained, in which the plant material extractions were performed. The Timatic Duo extraction system includes several key components:

− The extraction chamber, used in this study for processing dried and chopped plant samples;
− A control panel for adjusting extraction time and process pressure;
− An expansion vessel with a dual role: removing air from the system before extraction begins and ensuring solvent supply;
− A compressor that regulates pressure;
− Two hydropneumatic cylinders, a pump, and a collection vessel for the obtained extract.

The entire extraction system was adapted to operate under variable pressure conditions (both low and high). It was complemented with a Hielscher UP400St ultrasonic generator (Hielscher Ultrasonics GmbH, Teltow, Germany), directly connected to the extraction chamber. The role of ultrasound was to create a cavitation effect in the solvent, thereby facilitating the release of active compounds from the plants.

**Figure 4:**
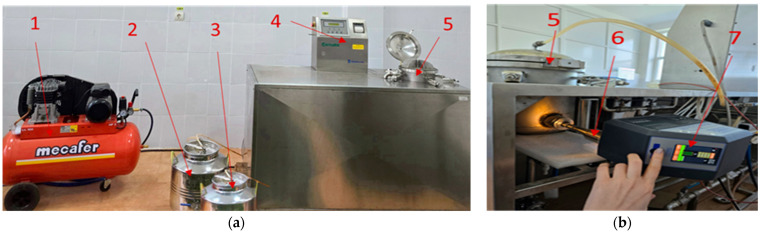
Hybrid pressure percolation–ultrasound extraction equipment: (**a**) front view; (**b**) back view. Components: 1—compressor, 2—expansion vessel, 3—extract collection vessel, 4—control panel, 5—extraction chamber, 6—sonotrode, 7—ultrasonic generator.

Water was used as the extraction solvent, chosen for its safety, availability, and compatibility with a sustainable process. The selection of this solvent contributed to both the safety and efficiency of the applied method (Figure 4).

In the preparation stage of the process, the permeable bags containing the dried and chopped raw material are introduced into the extraction chamber. Subsequently, the solvent (water) is transferred from the expansion vessel into the extraction chamber until it is completely filled. In addition, 7 L of buffer solvent (water) are used to fill the pipes and installation components, thus ensuring proper equipment operation. The process parameters—time and pressure—are set from the control panel, while ultrasound power is adjusted from the ultrasonic generator. After configuring all values, the equipment is started simultaneously, thereby initiating the extraction process. At the end of extraction, the extract is automatically collected in the collection vessel for subsequent analysis.

**Figure 5:**
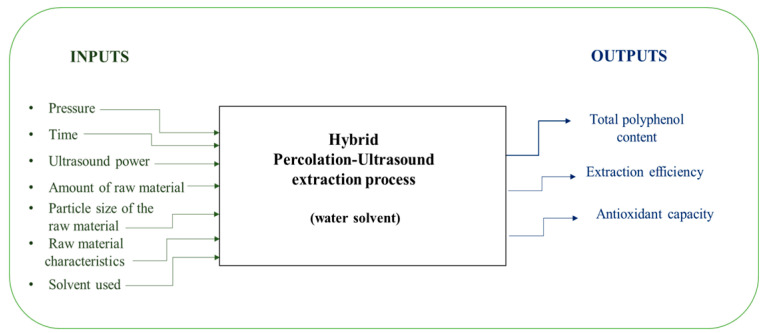
Systematic schematic of the extraction process of valuable components from aromatic plants through simultaneous percolation and sonication.

Figure 5 presents the general schematic of the extraction system together with the main input and output parameters used in the process. According to the literature, experimental designs involving more than two or three independent variables simultaneously can significantly complicate the process, making it more difficult to control and analyze, while also increasing workload and result instability. Considering these recommendations, as well as the technical limitations of the equipment used, a three-factor experimental design was selected, in which three independent variables (predictors) were analyzed: pressure (p, expressed in bar), extraction time (t, in minutes), and ultrasound power (P, in watts). The variation ranges of these factors are presented in Equation (1).

As the dependent variable (response), the total polyphenol content ($C_{p}$) obtained after extraction was evaluated. Within this system, it is assumed that functional relations exist between the dependent variable $C_{p}$ and the experimental factors p, t, and P, relations that will be analyzed in the following sections.

(1) $$C_{p}=C_{p}\left(p,\;t,\;P\right),\;p\in \left[5,7\right],\;t\;\in [60,\;120],\;P\in \;[80,\;100]$$

The experimental variables selection levels were based on some preliminary results of a previous study [12], which found that extraction at 140 W, 7 bar, and 60 min produced a lower polyphenol content compared with 120 W, 7 bar, and 60 min. Therefore, the study aimed to evaluate the combined effect of pressure, extraction time, and ultrasonic power on extraction efficiency, in order to identify the optimal configuration (Table 1). The maximum pressure tested (7 bar) was set by the percolator’s operating limit, and short extraction times were selected to maintain process efficiency and industrial relevance.

For each plant, 27 extraction samples were prepared, and each sample was performed and analyzed in triplicate. The obtained values were expressed as the mean of the technical replicates. Table 2 presents essential information about how the extraction and greenhouse studies were performed.

### 2.5. Determination of Polyphenol Content in Nettle and Sage Extracts

The total polyphenol content of the obtained extracts was determined using the Folin–Ciocalteu method, developed and validated in the laboratory based on the procedure described by [40]. For analysis, the extracts were diluted in a methanol–water mixture (1:1, *v*/*v*), and absorbance was measured with a UV–VIS spectrophotometer (Jasco V-550) (JASCO, Hachioji (Tokyo), Japan) at a wavelength of 755 nm. Polyphenol concentration was estimated from a calibration curve constructed using gallic acid as the standard. Results were expressed as milligrams of gallic acid equivalents per 100 g of dry plant material (mg GAE/100 g).

### 2.6. Determination of Antioxidant Capacity (DPPH—2,2′-Diphenyl-1-picrylhydrazyl) of Nettle and Sage Extracts

Antioxidant activity was evaluated using the DPPH method, which is based on the reduction of the stable radical 2,2′-diphenyl-1-picrylhydrazyl by antioxidant compounds in the sample. The radical scavenging capacity was expressed by the decrease in absorbance at 517 nm, measured spectrophotometrically using a UV–VIS spectrophotometer (Jasco V-550).

### 2.7. Presentation of Crops Treated with Biostimulant

#### 2.7.1. Soil Preparation Before Transplanting Tomato and Pepper Seedlings in the Greenhouse

The seedbed for tomato and pepper cultivation in the greenhouse was prepared by cleaning and loosening the soil to a depth of 30 cm using a Ruris 710 K power tiller (8.5 HP). A mixture of calcium sulfate (25–30 g/m^2^) and phosphorus fertilizer P 35 Bio Norofert (30–40 g/m^2^) was applied uniformly and incorporated into the upper 10–15 cm soil layer. Subsequently, irrigation was carried out with an EM-1 solution (10 mL/m^2^ diluted 1:100) to activate beneficial soil microorganisms. The seedbed was then left to rest for 3–5 days to allow microbial colonization. After leveling, planting rows were drawn, and healthy seedlings were transplanted at a spacing of 30–40 cm. This method improves root system development, nutrient availability, and plant resistance to diseases within an organic farming system (Table 3).

#### 2.7.2. Characterization of Vegetables Cultivated in the Greenhouse

Turda F1 is a sweet pepper hybrid with indeterminate growth, suitable for cultivation both in protected spaces (greenhouses and tunnels) and in open field conditions. The plants are vigorous and show good adaptability to various environmental conditions, ensuring high and consistent yields. The fruits have an average weight of 130–150 g. The Turda F1 hybrid has genetic resistance to *Cucumber mosaic virus* and *Tobacco mosaic virus*, contributing to improved yield stability.

Viva F1 is a tomato hybrid with indeterminate growth, suitable for cultivation both in polytunnels and in the open field. The plants are vigorous and well adapted to diverse growing conditions, showing good tolerance to stress. The fruits are large, weighing approximately 240–300 g. This hybrid is characterized by high yield potential, early maturation, and consistent productivity.

#### 2.7.3. Treatments with Biostimulant

For the biostimulant treatment, a mixture consisting of nettle and sage extracts (1:1) was used, obtained under the most efficient extraction conditions (pressure of 7 bar, ultrasound power of 120 W, and extraction time of 120 min). The extracts were combined in equal proportions and diluted with water, resulting in a final mixture at a ratio of 1:1 (water:plant extract). The biostimulant thus obtained was applied both foliar and at the root level, by irrigation, once per week in order to stimulate plant growth and to evaluate its effects on productivity.

#### 2.7.4. Cultivation of Tomato and Pepper Crops

Tomato and pepper crops were grown in a polytunnel measuring 10 m in width and 30 m in length, equipped with a ventilation system (three fans), shading net, and lateral openings for heat dissipation. The polytunnel was also fitted with a drip irrigation system, mulching film, and soil moisture sensors. During June and July, the climatic conditions inside the polytunnel were characterized by excessively high temperatures, which exceeded the capacity of the ventilation systems. As a result, it was necessary to open the structures to maintain an acceptable temperature, a measure that also facilitated pest entry and contributed to plant deterioration. Tomatoes were more affected by heat stress compared with peppers, mainly due to their larger vegetative mass, which is characteristic of the species and results in higher water loss through evapotranspiration. This water imbalance increases sensitivity to temperature fluctuations. The application of polyphenol-rich extracts exerted a protective effect by reducing oxidative stress.

#### 2.7.5. Quantification of Results (Chlorophyll, Growth Rate, Productivity)

To determine chlorophyll content in tomato and pepper plants cultivated in the polytunnel, for the purpose of evaluating the effect of the biostimulant, a Hansatech CL-01 (Hansatech Instruments Ltd, Pentney, King’s Lynn, Norfolk, UK) device was used. Representative plants were selected from each experimental variant (biostimulant-treated and control). Chlorophyll measurements were performed on ten plants per treatment (biological replicates, *n* = 10). On each plant, fully developed leaves located in the upper third were chosen, avoiding old or damaged leaves. For each leaf, 3–5 readings were taken in different areas, and the values were averaged. Measurements were performed on the same days and at the same time of day to avoid variations caused by light conditions. The results were centralized and statistically compared between variants to highlight the influence of the biostimulant on chlorophyll levels.

To determine the plant dry mass, samples consisting of three treated plants and three plants from the control group were collected for measurement. Weekly analyses were conducted throughout the development period of the two crops (tomatoes and peppers), with sampling performed on a weekly basis. After harvest, the plant material (leaves, stems, roots) was labeled and washed with distilled water to remove possible surface impurities. The samples were then placed in a drying oven (Caloris EC 25, Caloris Group SRL, Bucharest, Romania) at a constant temperature of 105 °C for 24 h, followed by placement in a desiccator for 2 h. Dry mass measurement was carried out using a precision analytical balance (AXS–KERN, Freimettigenstrasse 3, Konolfingen, Switzerland), and the results were expressed as grams per plant.

Productivity was evaluated using a methodology applied to a representative sample of 30 plants for each studied species (tomato and pepper), respectively, 15 treated plants and 15 control plants (*n* = 15 per treatment). Throughout the entire fruiting period, all fruits developed on the analyzed plants were harvested at regular intervals corresponding to the optimal harvest time specific to each species. Each fruit was individually weighed using a precision balance (AXS–KERN) to determine fresh weight. Thus, for each plant, the total fruit weight produced over the entire production cycle was calculated. At the same time, the total number of fruits formed per plant was recorded, allowing a detailed analysis of the relation between fruit number and total production weight.

The obtained data were centralized and statistically processed, and mean values were calculated for total fruit weight per plant, average number of fruits per plant, and average fruit weight. These parameters were used to compare the productivity of biostimulant-treated variants with that of untreated controls, contributing to the evaluation of the impact of biofertilization on plant production capacity.

## 3. Results

Vegetable crops established in greenhouses are frequently exposed to a combination of stress factors, both abiotic and biotic, which can negatively affect plant morphological development, physiological processes, and productivity. These include large temperature fluctuations, high humidity, insufficient light, salt accumulation in the substrate, as well as increased phytosanitary pressure caused by pathogen attacks.

In this context, our aim was to improve the extraction process of bioactive compounds from nettle and sage using a hybrid ultrasound-assisted percolation technology. Thus, the objective was to obtain extracts with a dual function: biofertilizing (by providing a balanced complex of macro- and microelements essential for plant metabolism) and bioprotective (due to the high content of polyphenolic compounds with antioxidant, antimicrobial, and plant defense response–stimulating activity).

### 3.1. Influence of Extraction Parameters on Total Polyphenol Content and Antioxidant Capacity

Table 4 highlights significant variations in the total polyphenol content of nettle and sage extracts, depending on the technological parameters used, namely, percolation pressure, process duration, and sonication power. The determination of polyphenol content and antioxidant activity was carried out as a method of characterizing the bioactive potential of the obtained solutions, considering the essential role of polyphenolic compounds in supporting the protective, adaptive, and metabolic stimulation functions of treated plants.

By correlating the polyphenol content with the determined antioxidant capacity, it is confirmed that the polyphenol classes extracted from nettle and sage are present in relevant concentrations in solution and possess significant biostimulant and bioprotective properties [41]. This relation is supported by the high values shown in the table, also indicating that the classes of polyphenols obtained in higher concentrations are responsible for the biological activity of the extract [29,30].

The highest polyphenol content was obtained at a pressure of 7 bar, a processing time of 120 min, and an ultrasound power of 120 W, these conditions also being associated with the highest antioxidant capacity of the extract.

### 3.2. Content of Micro- and Macronutrients in Sage and Nettle Extracts Using Hybrid Percolation–Ultrasound Technology

The hybrid percolation–ultrasound extraction technology proved effective in obtaining bioprotective and biofertilizing solutions due to the synergy between the controlled diffusion of soluble compounds through percolation and the mechanical disruption of cell walls induced by ultrasonic cavitation, which results in an increased and selective release of phenolic compounds and essential nutrients. In addition, the selected parameters aimed to maintain the temperature within predefined ranges that prevent the thermal degradation of sensitive bioactive compounds in the two analyzed plant species. Although temperature was not an actively controlled parameter, it was continuously monitored with a temperature sensor and did not exceed 35 °C. Table 5 presents the values obtained for micro- and macronutrients under the extraction mode considered optimal.

The high concentrations of micro- and macronutrients support the applicability of nettle and sage extracts as integrated solutions for treatments applied to greenhouse vegetable crops. The composition of the nettle extract recommends it for stimulating vegetative growth and supporting physiological and fruiting processes. On the other hand, the sage extract contributes to the induction of systemic plant resistance and to the reduction of oxidative stress effects, playing an important role in protection against diseases and harmful abiotic factors (due to its polyphenol content).

### 3.3. Development of a Biofertilizing and Bioprotective Solution by Combining Salvia officinalis and Urtica dioica Extracts Obtained Through Percolation–Ultrasound, and Validation of the Results on Tomato and Pepper Crops

**Figure 6:**
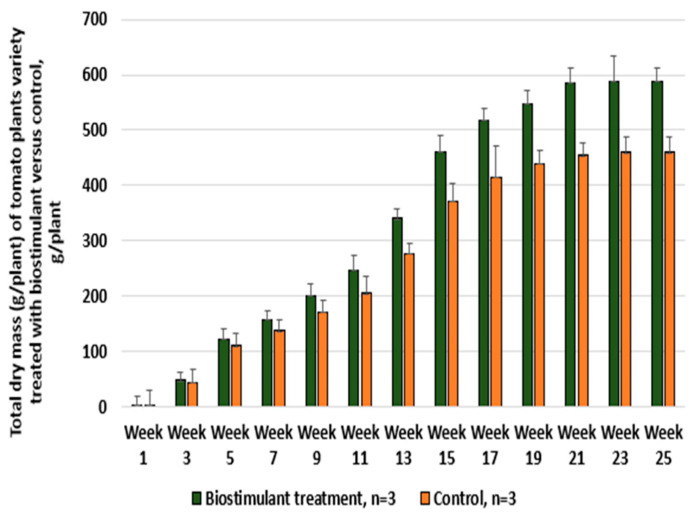
Weekly comparative analysis of total dry mass for tomato plants treated with biostimulant versus untreated control.

**Figure 7:**
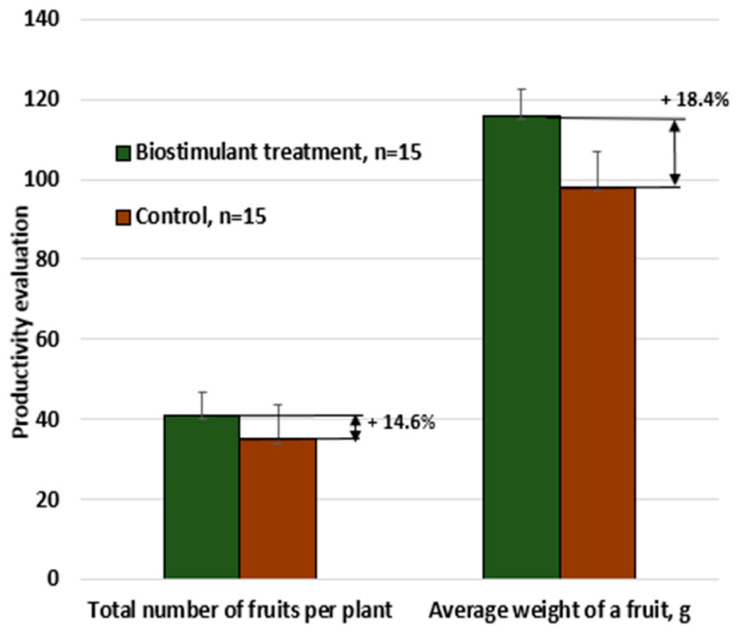
Productivity evaluation of tomato plants treated with biostimulant versus untreated control.

By combining the two extracts (mixed at a 1:1 ratio), a synergistic effect is obtained that supports both balanced plant nutrition and the enhancement of natural defense capacity, providing a sustainable and effective alternative to conventional agrochemicals. The comparative evaluation of tomatoes treated with the biostimulant versus the control sample is presented in Figure 6 with respect to dry mass per plant, collected weekly, and in Figure 7, which illustrates the increase in productivity in terms of the number of fruits per plant and the average fruit weight.

For tomatoes, the total productivity increase (resulting from both the higher number of fruits and the increase in average fruit weight) was 34–38% per plant.

**Figure 8:**
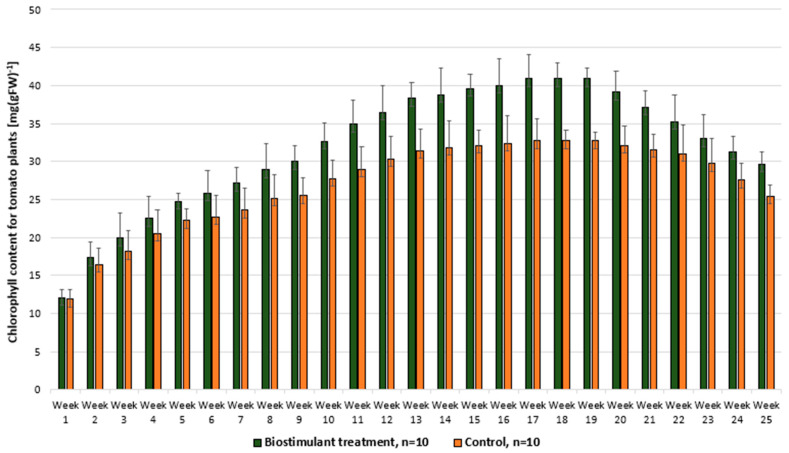
Comparative evaluation of chlorophyll content in tomato plants treated with biostimulant versus untreated control.

According to Figure 8, the chlorophyll content of Viva F1 tomato leaves is influenced by the developmental stage of the plant, showing higher values during active growth phases and a tendency to decrease as the plant reaches maturity, a process associated with the progressive reduction of chlorophyll content in leaves.

**Figure 9:**
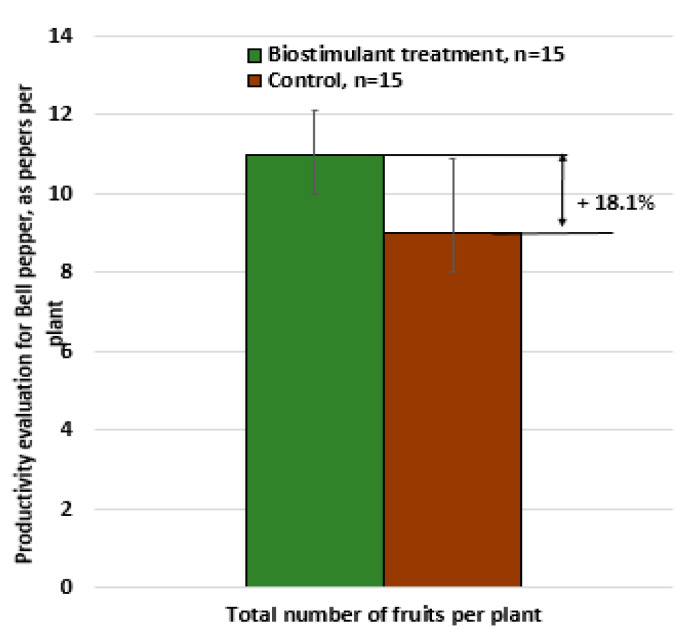
Productivity assessment of bell pepper: number of marketable fruits per plant—biostimulant treatment vs. untreated control.

**Figure 10:**
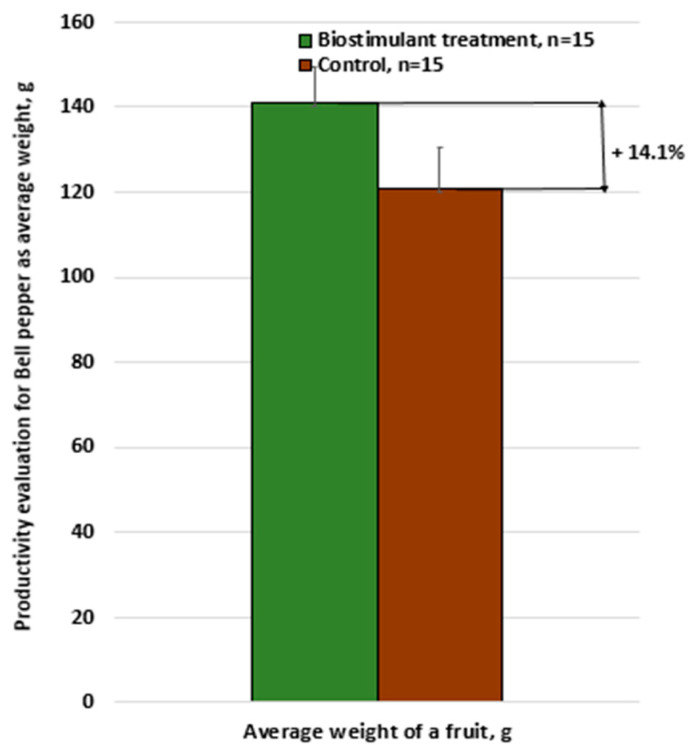
Productivity assessment of bell pepper: average fruit weight—biostimulant treatment vs. untreated control.

**Figure 11:**
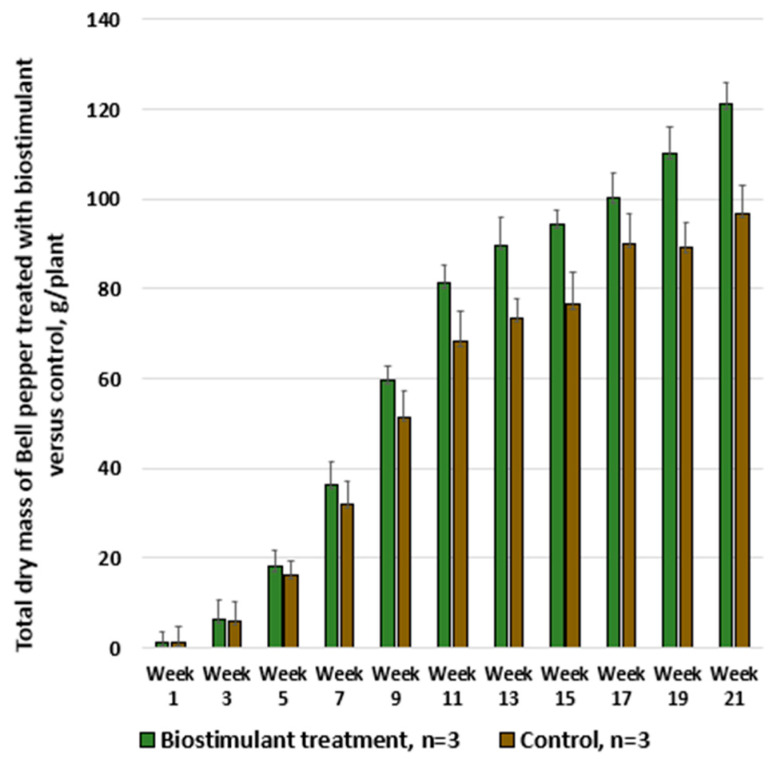
Weekly comparative analysis of total dry mass in Bell pepper plants treated with biostimulant versus untreated control.

Similarly, tests were also performed on the greenhouse-grown bell pepper cultivar Turda F1, with the results illustrated in Figure 9, Figure 10 and Figure 11.

In this case as well, the differences were significant, with each bell pepper plant treated with the biostimulant producing 18% more compared with untreated plants (the cumulative mean effect resulting from both the increase in fruit number and the increase in fruit weight).

**Figure 12:**
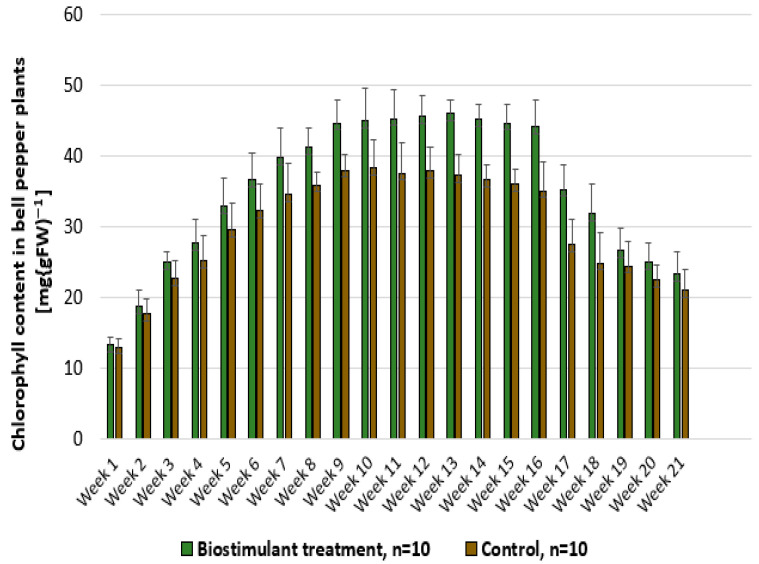
Comparative evaluation of chlorophyll content in bell pepper plants treated with biostimulant versus untreated control.

In Figure 12, it can be observed that the chlorophyll content in bell pepper leaves gradually increased during the first weeks and reached maximum values between weeks 9 and 14, a period corresponding to the phase of intensive fruiting and the onset of harvesting. Treatment with biostimulant maintained a higher chlorophyll content compared with the control, supporting more active photosynthesis throughout the plant development period. After week 15, as the plants directed resources toward fruit maturation, the chlorophyll content began to decline. During weeks 19–21, the difference between measurements decreased, as the impact of the treatment was lower in this late stage when the plants were approaching the end of the production cycle.

**Figure 13:**
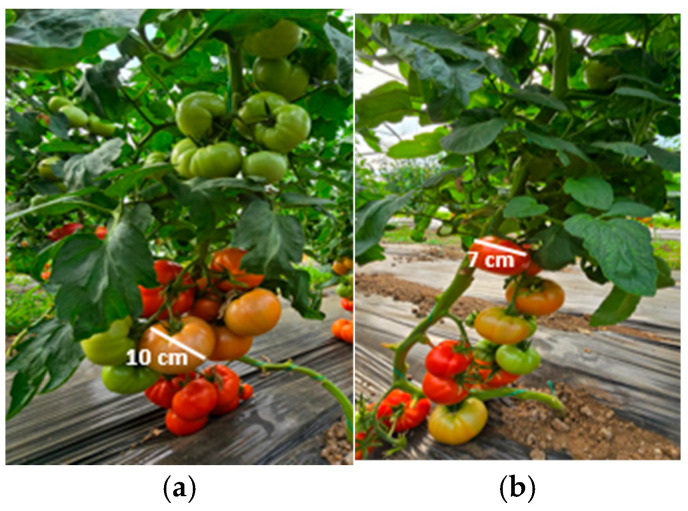
Productivity differences in tomato: (**a**) plants treated with biostimulant; (**b**) untreated control.

**Figure 14:**
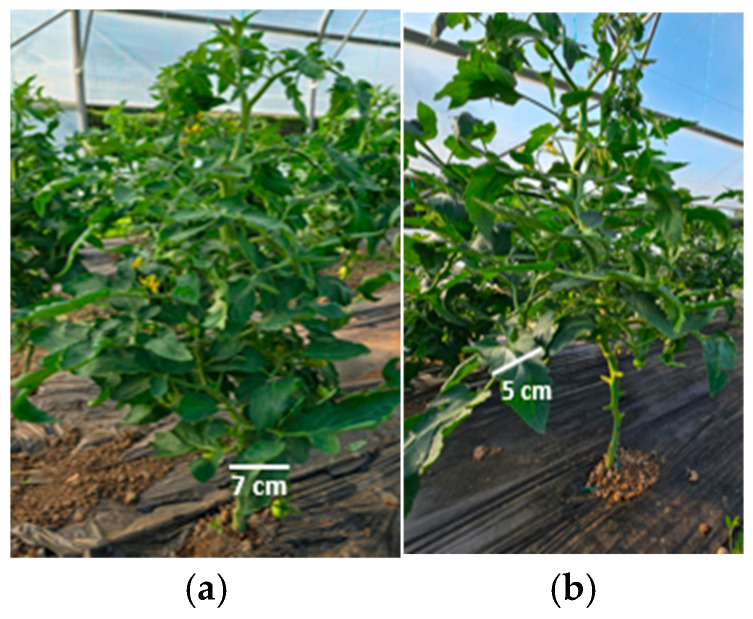
Comparative evaluation of vegetative biomass in tomato: (**a**) plants treated with biostimulant; (**b**) untreated control.

**Figure 15:**
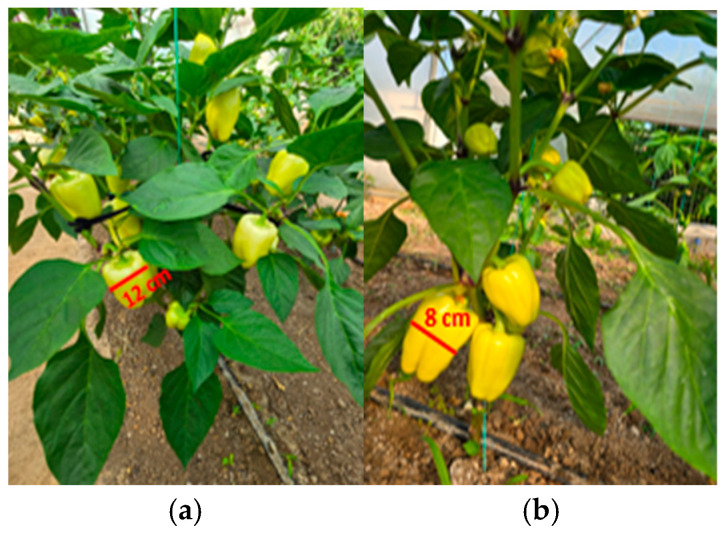
Productivity differences in bell pepper: (**a**) plants treated with biostimulant; (**b**) untreated control.

**Figure 16:**
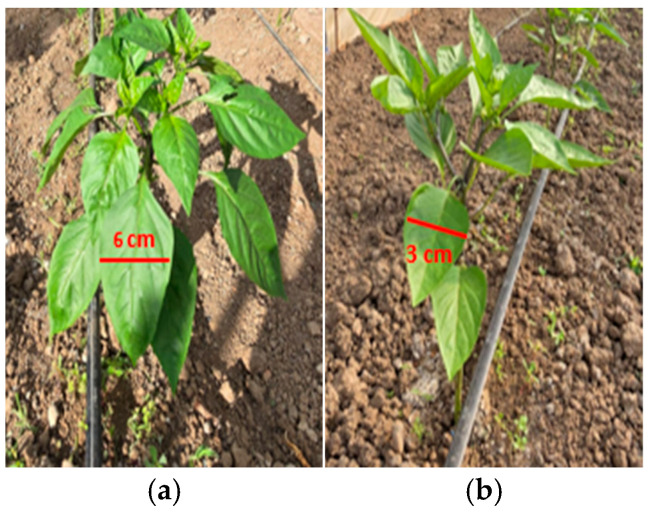
Comparative evaluation of vegetative biomass in bell pepper: (**a**) plants treated with biostimulant; (**b**) untreated control.

**Figure 17:**
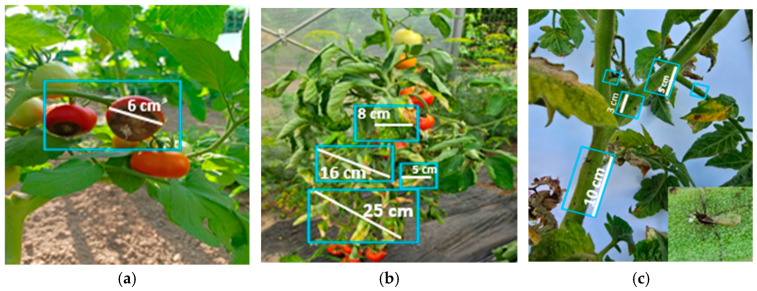
Incidence of diseases and pests observed in untreated tomato plants: (**a**) blossom end rot; (**b**) tomato mosaic virus (ToMV); (**c**) pest *Myzus persicae* syn. *Aphis persicae* (alate form).

**Figure 18:**
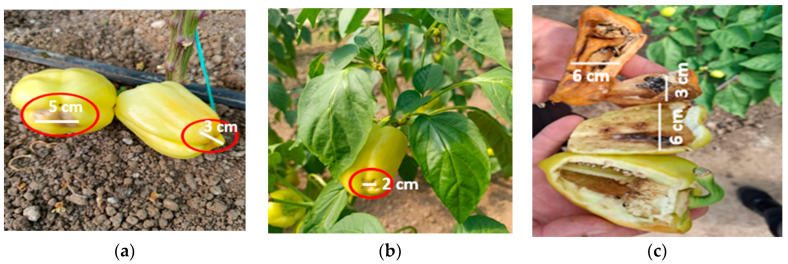
Incidence of diseases and pests observed in untreated bell pepper plants: (**a**,**b**) blossom end rot; (**c**) pest *Helicoverpa armigera*.

To complete the analysis, Figure 13, Figure 14, Figure 15, Figure 16, Figure 17 and Figure 18 present a comparison between tomato and bell pepper plants and fruits from the biostimulant-treated variants and the control, highlighting morphological differences and allowing a visual evaluation of the effects of the applied treatments.

In the case of tomatoes (Figure 13 and Figure 14), the fruits from treated plants were more numerous, larger in size, and the plants exhibited higher vegetative density with a greater number of leaves. Similar results were observed for bell pepper (Figure 15 and Figure 16), where biostimulant treatments led to more vigorous plant development and higher yield.

In untreated tomato crops, symptoms of blossom end rot caused by functional calcium deficiency in the fruit were observed, along with signs of tomato mosaic virus (ToMV), manifested through deformed and mosaic-patterned leaves, as well as the presence of the aphid *Myzus persicae*. This pest not only weakens plants by piercing-sucking activity but also contributes to the transmission of viral diseases. Aphid infestation leads to leaf yellowing, deformation, and drying, reduced photosynthesis, and consequently decreased plant vigor and productivity (Figure 17).

In untreated control plants, typical spots of blossom end rot appeared, caused by a functional calcium deficiency in the fruit. In contrast, plants treated with nettle and sage extracts showed fewer or less severe symptoms, suggesting a possible positive influence on plant physiological status. However, no microbiological or nutrient analyses were performed to confirm the mechanism, and further studies are required. The presence of the pest *Helicoverpa armigera* caused holes in the fruits and pulp destruction, which led to reduced fruit quality as well as an increased risk of disease occurrence (Figure 18).

## 4. Discussion

### 4.1. Analysis of the Extract’s Composition Obtained from Nettle (Urtica dioica) and Sage (Salvia officinalis)

The analysis of the extract’s composition highlights the complementarity of the two plants in terms of potential functional roles within protected agroecosystems. The nettle (*Urtica dioica*) extract contains significant levels of essential macronutrients as well as important concentrations of trace elements involved in enzymatic activity, making it an effective biofertilizing agent. These elements play a direct role in fundamental metabolic processes such as photosynthesis, protein synthesis, root development, and osmotic regulation, thereby supporting accelerated vegetative growth, enhanced flowering, and improved fruit quality [42,43]. On the other hand, sage (*Salvia officinalis*) extract is distinguished by its superior phytochemical profile, particularly with respect to its total polyphenol content (up to 30.79 mg GAE/100 g) and high antioxidant capacity (up to 85.61 mg Trolox/100 g), underlining the strong bioprotective potential of this plant extract. Polyphenolic compounds are known for their antimicrobial, antifungal, and plant defense-inducing properties, and may reduce the incidence of common fungal and bacterial diseases in tomato and pepper crops, such as powdery mildew, early blight, or bacterial spot, while also exerting protective effects against pests [30,31,44]. Therefore, the combined use of the two extracts produces a synergistic effect, simultaneously fulfilling the plants’ essential nutritional requirements and strengthening their natural resistance to both biotic and abiotic stresses—factors of critical importance in protected cultivation systems (greenhouses and high tunnels).

The optimization of extraction parameters was designed to maximize the Total Polyphenol Content (TPC), chosen as the key indicator of the extracts’ antioxidant and bioprotective potential. This target aligns with the industrial objective of developing bulk functional materials for sustainable agriculture, where higher TPC values translate into enhanced bioactivity and improved plant resilience.

The mineral composition of nettle and sage extracts shows relevant levels of potassium, calcium, and magnesium, which are known to support essential plant functions. Potassium regulates stomatal opening and improves water and nutrient transport within the plant. Calcium strengthens cell walls and contributes to stress resistance, while magnesium activates key enzymes involved in photosynthesis. Also, the phenolic compounds protect plant cells from oxidative stress and help the plant resist disease and environmental stress [45,46]. Similar effects have been reported, showing increased chlorophyll content and photosynthetic activity [47], which contribute to higher yield and improved plant quality in treated crops [42,43].

### 4.2. Mathematical Assessment of the Relationship Between Processing Parameters and Polyphenol Yield in Nettle and Sage Extracts

The main objective of the mathematical modeling was to identify the functional relationships and the influence of each processing parameter—pressure, ultrasound power, and processing time—on the extraction yield, expressed as the total amount of polyphenols obtained. By developing a predictive model, the study aimed to improve the extraction process and achieve a better understanding of the underlying extraction mechanism.

**Figure 19:**
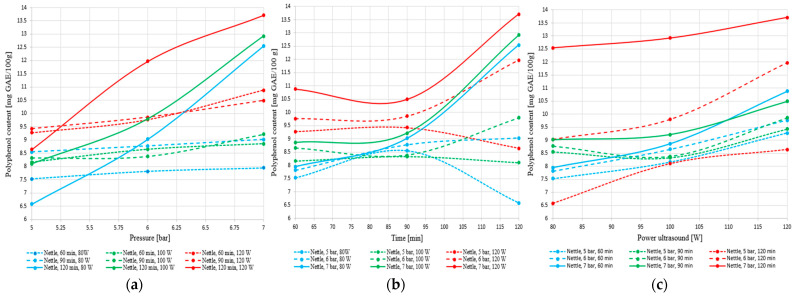
Variation of total polyphenol content in nettle extract as a function of (**a**) pressure, (**b**) extraction time, and (**c**) ultrasound power.

The graphs presented in Figure 19a–c illustrate the influence of pressure, processing time, and ultrasound power on the total polyphenol content extracted from nettle.

The graphical analysis of the influence of pressure, extraction time, and ultrasound power on the total polyphenol content in nettle reveals a general trend of increasing yield with the intensification of processing conditions. Pressure exerts a clearly positive effect, particularly at longer extraction times and higher ultrasonic power, while ultrasound power significantly contributes to the release of polyphenolic compounds through cavitation effects.

Higher percolation pressure improved solvent penetration into the plant matrix and supported the release of soluble compounds, which is consistent with the higher polyphenol levels obtained at 7 bar. Ultrasound further enhanced extraction through cavitation, which disrupts plant cell structures and increases the availability of intracellular polyphenols. This effect aligns with the increase recorded at 120 W, where cavitation contributed to a higher extraction yield. The proposed extraction technology combines the use of pressure and ultrasound accelerated mass transfer, in order to obtain the highest total polyphenol content. The tests performed confirm the synergistic action of these two processes in improving extraction efficiency, with 7 bar, 120 W, and 120 min identified as the most effective operating conditions.

Extraction time has a positive influence up to a certain point, after which efficiency plateaus or even declines, especially under suboptimal conditions. The highest polyphenol concentration was obtained at 7 bar, 120 min, and 120 W. These results confirm the need for a multifactorial approach to improve the extraction process.

**Figure 20:**
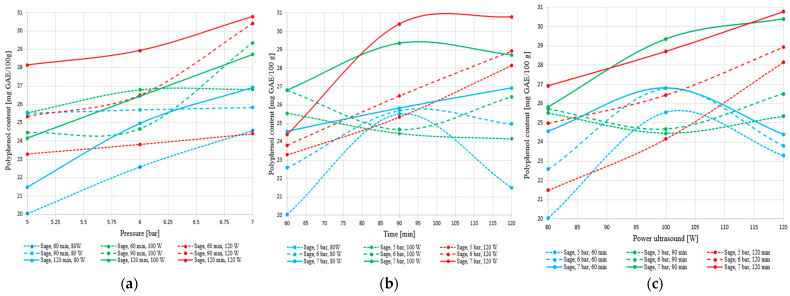
Variation of total polyphenol content in sage extract as a function of (**a**) pressure, (**b**) extraction time, and (**c**) ultrasound power.

Figure 20a–c illustrate the influence of pressure, extraction time, and ultrasound power on the total polyphenol content extracted from sage.

The results obtained for sage confirm the superior performance of this species in hybrid extraction, yielding the highest polyphenol levels compared to nettle (over 31 mg GAE/100 g). The plant responds positively to increases in pressure, extraction time, and ultrasound power, showing a clear upward trend in yield under intensive conditions (7 bar, 120 min, 120 W). Unlike nettle, sage does not exhibit signs of plateauing or degradation within the tested range. Although at shorter extraction times the relationship with acoustic power is slightly parabolic, at 120 min the behavior becomes stable and efficient, indicating a controlled and sustained release of phenolic compounds.

### 4.3. Correlation Matrix for Nettle and Sage

The correlation matrix between the process variables considered in the experiments was obtained using the PTC Mathcad Prime 9.0.0.0 program [48] and the Statistics Kingdom web application—Multiple Linear Regression Calculator [49], the results being presented in Table 6.

It was observed that the independent variables are uncorrelated, while the dependent variable (*Cₚ*) shows significant correlation with all independent variables. Specifically, $C_{p}$ is most strongly and directly correlated with pressure $\left(p\right)$, extraction time $\left(t\right)$, and ultrasound power $\left(P\right)$. Overall, $C_{p}$ exhibits a positive correlation with all the independent variables.

### 4.4. Results of Linear Multivariate Analysis for Nettle

The structural dependence functions for the significance level settings α = 0.05 (effect size = 0.39, medium effect type f, [48] program settings are given in Equations (2) and (3).

(2) $$C_{p}=-4.479037+1.166p+0.02665t+0.0450361P$$

The results of the multiple linear regression showed a significant collective effect of the independent parameters (predictors) p, t, P, and the dependent parameter, $C_{p}$. The obtained coefficient of determination R^2^ = 0.669, as well as the adjusted coefficient of determination, R^2^_adj_ = 0.63, confirm the above finding. The independent variables p, t, P, explain 66.9% of the variation in $C_{p}$.

### 4.5. Results of Multivariate Polynomial Regression Statistics for Nettle

Using polynomial regression statistics, more general results were obtained compared to those provided by the multiple linear regressions in Section 4.4. These regressions were calculated with the function available in the Mathcad 15 program, as referenced in [49]. In ascending order of the degrees of the interpolation polynomials, the following values were obtained (Table 7).

It was observed that for the complete second-degree polynomial, many of the calculated probabilities of ignoring the coefficients exceed 0.05. These values indicate that polynomial interpolation is reliable only for first-degree polynomials. However, several terms of the complete second-degree polynomial are either below the 0.05 significance threshold or only slightly above it; such terms were therefore retained in the second-degree polynomial regression selected by the multivariate regression analysis program. For the second-degree progression, with coefficients listed in Table 6, the coefficient of determination was 0.889, while the adjusted coefficient of determination was 0.83. The nonlinear regression given in Equation (3) was thus obtained. In this case, the statistical analysis program [48] selected the final second-degree polynomial model as expressed in Equation (3).

(3) $$C_{p}=17.371707-2.11475p-0.192067t+0.0364528pt+0.000227666P^{2}$$

The coefficient of determination reached R^2^ = 0.86, while the adjusted coefficient of determination was R^2^adj = 0.84. The predictors considered in the nonlinear regression (3), p, t, pt and P, explain 86.4% of the variation in $C_{p}$, representing a substantial improvement over the linear regression (2).

Another nonlinear regression, proposed in [49], is the power regression presented in Formula (4).

(4) $$C_{p}=0.121781p^{0.7014}t^{0.20496}P^{0.472451}$$

In this case, the coefficient of determination was R^2^ = 0.67, while the adjusted coefficient of determination was R^2^adj = 0.62. The predictors of regression (4) account for 66.8% of the variation in the dependent variable $C_{p}$.

### 4.6. Minimum and Maximum Values of the Linear Statistical Model of Polyphenol Content for Nettle

The minimum and maximum values of the $C_{p}$ function with three arguments, as defined by Formula (2), were calculated using the numerical minimization and maximization functions described in source [49]. For this case (multivariate linear regression of polyphenol content in nettle extract), the corresponding minimum and maximum values are presented in Table 8.

### 4.7. Graphical Representations for Nettle

**Figure 21:**
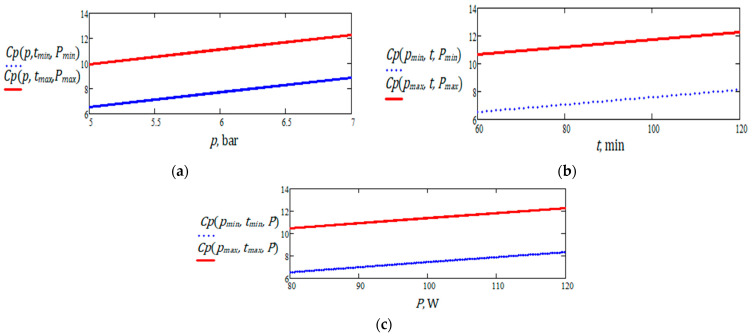
Graphic representation of polyphenol content for nettle: (**a**) as a function of pressure, at minimum and maximum levels of extraction time and ultrasound power; (**b**) as a function of extraction time, for minimum and maximum values of ultrasound pressure and power; (**c**) as a function of ultrasound power, for minimum and maximum values of pressure and extraction time.

Several graphical representations of partial functions with two and one variables, describing the polyphenol content, are shown in Figure 21. These illustrate the evolution of the response as a function of a single variable. It can be observed that the minimum and maximum polyphenol values are located at the boundaries of the investigated experimental domain.

The graphical representations show that, within the limited ranges of variation related to the three independent variables and under the assumptions of the linear model, the polyphenol content (dependent variable) exhibits a monotonic increase in relation to the independent variables (nettle).

### 4.8. Results of Multivariate Linear Analysis for Sage

The structural dependence functions, determined at a significance level of α = 0.05 (effect size = 0.39, medium effect type f, program parameters according to source [48]), are presented in Formulas (5) and (6).

(5) $$C_{p}=5.394167+1.656444p+0.0420852t+0.0666361P$$

The results indicate that the independent variables p, t and P are highly significant predictors of the dependent variable $C_{p}$. The coefficient of determination (R^2^ = 0.65) and the adjusted coefficient of determination (R^2^adj = 0.60) reveal that these variables collectively account for approximately 62.6% of the variation in $C_{p}$. All predictors included in the model are statistically significant for the dependent variable, with p, t and P explaining about 65% of the variation in the polyphenol content ($C_{p}$).

### 4.9. Results of Multivariate Polynomial Regression Analyses for Sage

The use of polynomial regression provides more general results compared to the multiple linear regression analysis that was presented in Section 4.8. Arranged in ascending order according to the degree of the interpolation polynomials, the results are summarized in Table 8. It can be observed that, for the complete second-degree polynomial, the probability values associated with coefficient exclusion exceed 0.05, indicating that polynomial interpolation is reliable only for first-degree polynomials. According to the program described in source [48], the second-degree terms pt, tP and $t^{2}$ are retained following the multilinear regression analysis. The resulting nonlinear regression model (6) achieves a coefficient of determination of R^2^ = 0.74 and an adjusted coefficient of determination of R^2^adj = 0.70 (F(3,23) = 21.42).

(6) $$C_{p}=15.831455+0.0184703pt+0.000796597tP-0.000826513t^{2}$$

It can be observed that the multivariate regression analysis program selects the final model based on the coefficients with the lowest probability of rejection, as shown in the complete results provided in source [49], Table 8. The program described in source [48] reports results for each computational step, whereas the results presented in Table 9 (source [49]) correspond to the initial step of the multivariate analysis. For the linear regression model obtained with the first-degree interpolation polynomial (source [49]), the coefficient of determination is R^2^ = 0.65, with an adjusted value of R^2^adj = 0.604. For the second-degree interpolation polynomial, the coefficient of determination increases to R^2^ = 0.776, with an adjusted value of R^2^adj = 0.658.

Another nonlinear regression suggested by [49] is the power regression in Equation (7).

(7) $$C_{p}={2.044269p}^{0.382214}t^{0.145835}P^{0.260955}$$

For this model, the coefficient of determination is R^2^ = 0.66, with an adjusted coefficient of determination of R^2^adj = 0.61. The F-test yields F(3,23) = 14.81, *p* < 0.001. The shredding degree parameter is not significant. Overall, the predictors included in regression model (7) account for 65.9% of the variation in the dependent variable $C_{p}$.

### 4.10. Minimum and Maximum Values of the Linear Statistical Model for Polyphenol Content for Sage

The minimum and maximum values of the $C_{p}$ function, with three arguments, given by Formula (5), is calculated using the numerical minimization and maximization functions of the source [49]. The minimum and maximum values are given, for this case (multivariate linear regression of polyphenol content in the case of extraction from sage plants), in Table 10.

### 4.11. Graphical Representations for Sage

**Figure 22:**
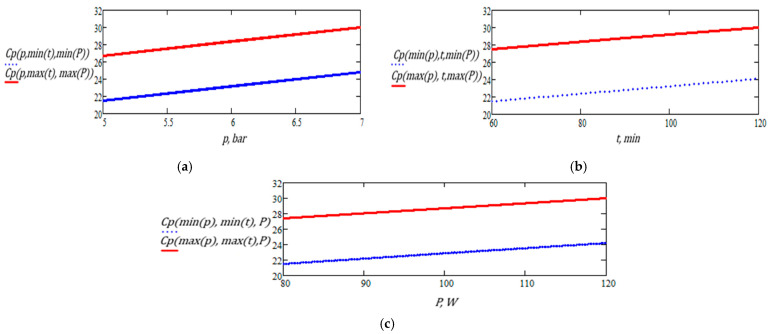
Graphic representation of polyphenol content for sage: (**a**) as a function of pressure, at minimum and maximum levels of extraction time and ultrasound power; (**b**) as a function of extraction time, for minimum and maximum values of ultrasound pressure and power; (**c**) as a function of ultrasound power, for minimum and maximum values of pressure and extraction time.

Figure 22 presents graphical representations of partial functions of two and one variable for the total polyphenol content extracted from the sage plant. The figures in this subchapter illustrate one-dimensional representations. It is observed that the extreme values of polyphenol content occur at the boundaries of the working domain, corresponding to the combinations (*pmin*, *tmin*, *Pmin*) and *(pmax*, *tmax*, *Pmax*). This distribution aligns with the expected behavior: beyond a certain extraction duration, the polyphenol reserves in the plant material are progressively depleted, and the incremental effects of pressure and ultrasound diminish. In this region, nonlinear effects may emerge due to the functional limits of the process, both temporally and within the three-dimensional variable space $\left(p,\;t,P\right)$. A more detailed exploration of these effects would require expanding the experimental domain and applying a finer discretization of the variables to characterize the saturation region more precisely.

The graphical representations indicate that, within the limited working intervals of the three independent variables and under the constraints of the linear model, the polyphenol content (dependent variable) increases monotonically with the independent variables for sage plant.

Although this study provided relevant results, there are aspects that require further investigation. The experiments were carried out under controlled conditions and included a limited number of plant species and extraction parameters. It is necessary to extend the application of the hybrid method to other medicinal plants with different phytochemical profiles in order to evaluate its potential to obtain extracts rich in bioactive compounds. Assessing the influence of other technological variables, such as solvent temperature, solvent type, or the number of extraction cycles, may contribute to further optimization of the polyphenol recovery process. From an agricultural perspective, testing the biostimulant on more crops and under field conditions is necessary to confirm its effectiveness in practice. Evaluating different doses and application frequencies may help establish the most efficient way to use it in agriculture.

## 5. Conclusions

The hybrid extraction technology based on ultrasound-assisted percolation proved to be particularly effective for obtaining bioactive compounds from sage and nettle, with applications in vegetable cultivation. The optimal operational extraction parameters identified in the evaluations were as follows: pressure 7 bar, ultrasound power 120 W, extraction time 120 min, and particle size 30 mm.

The biostimulant solutions were tested on pepper and tomato plants to evaluate their growth-stimulating effects. Treated plants showed higher productivity (38–53%) and increased total dry mass (up to 22%) compared to controls. The polyphenolic compounds and bioactive constituents in the biostimulant extracts contributed to improved plant performance, highlighting their potential as sustainable plant-based biostimulant treatments.

The results of the statistical and compositional analysis demonstrate that process parameters significantly influence the extraction mechanism, highlighting relevant correlations between technological variables. The modeling performed supports the possibility of more efficient process control and confirms that the hybrid method represents a viable, efficient, and sustainable option for obtaining plant extracts applicable to crop cultivation in protected environments.

A limitation of the present research is that the analysis is valid only for the plant subspecies used in these experiments, under the cultivation and harvesting conditions specified in this study.

## Figures and Tables

**Table 1:** Parameters of the hybrid extraction process.

Time	Pressure	Power	Chopping Degree
min	bar	W	cm
60	5	80	3
60	6	80	3
60	7	80	3
60	5	100	3
60	6	100	3
60	7	100	3
60	5	120	3
60	6	120	3
60	7	120	3
90	5	80	3
90	6	80	3
90	7	80	3
90	5	100	3
90	6	100	3
90	7	100	3
90	5	120	3
90	6	120	3
90	7	120	3
120	5	80	3
120	6	80	3
120	7	80	3
120	5	100	3
120	6	100	3
120	7	100	3
120	5	120	3
120	6	120	3
120	7	120	3

**Table 2:** Summary of the main experimental parameters for extraction and greenhouse trials.

Parameter Category	Parameter	Value/Description
Plant material	Species	Nettle (*Urtica dioica*) Sage (*Salvia officinalis*)
	Plant part used	Leaves, stems, flowers (dried)
	Particle size	30 mm
Solvent and Extraction Setup	Solvent	Water
	Solvent volume	12 L
	Plant material	400 g
	Solid to liquid ratio	1:30 (*w*/*v*)
	Extraction technique	Hybrid ultrasound-assisted percolation
	Pressure	5, 6, 7 bar
	Extraction time	60, 90, 120 min
	Ultrasound power	80, 100, 120 W
	Replicates (technical)	3 per sample
Greenhouse trials	Crop species	Tomato, pepper
	Treatments	Biostimulant vs. Control
	Biological replicates	100 treated + 100 control per crop
	Plant layout	Plants arranged in rows
	Parameters measured	Chlorophyll, total dry mass, productivity
Statistical analysis	Statistical design	Randomization was used to ensure plants were assigned to the treatment groups by chance

**Table 3:** Physico-chemical analyses of soil.

Sample	Sample Depth (cm)	Analyses Performed						
		pH (pH Units)	C_org_ (% su)	Humus (% su)	N (% su)	P_AL_ (mg/kg su)	K_AL_ (mg/kg su)	Total Soluble Salts (mg/100 g)
P1	2–30	6.97	3.46	5.95	0.320	151	212	158
P2	2–30	6.82	3.45	5.93	0.370	112	198	178
P3	2–30	7.34	3.43	5.91	0.365	132	203	210
P4	2–30	7.03	3.47	5.96	0.391	145	205	201
P5	2–30	7.06	3.48	6.01	0.410	177	186	199

**Table 4:** Total polyphenol content and antioxidant activity of nettle and sage extracts as a function of extraction parameters.

Pressure (bar)	Time (min)	Power (W)	Total Polyphenol Content (mg GAE/100 g)		Antioxidant Capacity of Extracts (mg Trolox/100 g)	
			Nettle	Sage	Nettle	Sage
5	60	80	7.53 ± 0.161	20.04 ± 0.52	15.87 ± 0.277	72.76 ± 1.66
6	60	80	7.82 ± 0.204	22.59 ± 0.58	16.21 ± 0.316	72.88 ± 1.76
7	60	80	7.95 ± 0.249	24.57 ± 0.537	16.50 ± 0.379	73.69 ± 1.286
5	60	100	8.16 ± 0.343	25.55 ± 0.604	17.29 ± 0.476	75.47 ± 1.375
6	60	100	8.65 ± 0.262	26.79 ± 0.704	18.54 ± 0.422	78.05 ± 1.565
7	60	100	8.87 ± 0.323	26.81 ± 0.693	19.15 ± 0.448	78.71 ± 1.507
5	60	120	9.28 ± 0.314	23.29 ± 0.719	20.91 ± 0.428	73.01 ± 1.580
6	60	120	9.77 ± 0.199	23.81 ± 0.624	21.44 ± 0.326	73.12 ± 1.750
7	60	120	10.88 ± 0.203	24.40 ± 0.661	22.66 ± 0.331	73.33 ± 1.832
5	90	80	8.56 ± 0. 188	25.51 ± 0.685	18.30 ± 0.321	74.93 ± 1.943
6	90	80	8.78 ± 0.204	25.70 ± 0.565	18.33 ± 0.374	75.97 ± 1.733
7	90	80	9.02 ± 0.239	25.83 ± 0.695	19.69 ± 0.378	76.10 ± 1.937
5	90	100	8.33 ± 0.216	24.45 ± 0.673	17.76 ± 0.597	73.41 ± 1.868
6	90	100	8.39 ± 0.212	24.66 ± 0.718	18.10 ± 0.570	73.85 ± 2.004
7	90	100	9.22 ± 0.165	29.35 ± 0.770	20.78 ± 0.538	82.11 ± 2.084
5	90	120	9.43 ± 0.226	25.34 ± 0.624	21.20 ± 0.604	74.68 ± 1.781
6	90	120	9.86 ± 0.196	26.50 ± 0.555	21.70 ± 0.559	77.59 ± 1.725
7	90	120	10.50 ± 0.360	30.40 ± 0.638	22.17 ± 0.556	83.27 ± 1.770
5	120	80	6.59 ± 0.229	21.49 ± 0.611	14.79 ± 0.481	72.80 ± 1.699
6	120	80	9.03 ± 0.272	24.97 ± 0.634	20.06 ± 0.488	74.02 ± 1.723
7	120	80	12.55 ± 0.294	26.93 ± 0.595	23.31 ± 0.535	78.81 ± 1.690
5	120	100	8.10 ± 0.299	24.16 ± 0.675	16.95 ± 0.537	73.22 ± 1.816
6	120	100	9.80 ± 0.226	26.44 ± 0.617	21.58 ± 0.488	76.98 ± 1.721
7	120	100	12.92 ± 0.204	28.72 ± 0.662	23.79 ± 0.462	81.23 ± 1.782
5	120	120	8.64 ± 0.239	28.15 ± 0.646	18.42 ± 0.510	80.02 ± 1.779
6	120	120	11.97 ± 0.230	28.94 ± 0.730	23.05 ± 0.489	81.85 ± 1.878
7	120	120	13.71 ± 0.211	30.79 ± 0.760	24.15 ± 0.486	85.61 ± 2.140

**Table 5:** Nutrient elements of interest determined in sage and nettle extracts using hybrid percolation–ultrasound technology.

	Plant	N	P	Ca	Mg	K	Zn	Mn	Fe	Cu	MU
Chemical analysis of the plant	sage	19.6 ± 0.104	1.9 ± 0.115	12.79 ± 0.201	4.72 ± 0.153	16.02 ± 0.108	22.01 ± 0.211	76.89 ± 0.161	405.9 ± 0.113	9.92 ± 0.199	mg/g
	nettle	48.16 ± 0.117	5.4 ± 0.256	31.06 ± 0.109	3.67 ± 0.110	41.59 ± 0.142	31.49 ± 0.128	42.9 ± 0.139	205.4 ± 0.111	22.65 ± 0.124	
Chemical analysis of the obtained extract	sage	38 ± 0.211	20.96 ± 0.194	102.07 ± 0.203	68.14 ± 0.101	405 ± 0.195	0.23 ± 0.187	0.49 ± 0.166	0.012 ± 0.181	0.06 ± 0.201	mg/L
	nettle	123 ± 0.139	64.41 ± 0.271	170.1 ± 0.208	40.9 ± 0.193	1003 ± 0.136	0.57 ± 0.117	0.1 ± 0.170	0.003 ± 0.118	0.143 ± 0.213	

MU = measurement unit.

**Table 6:** Correlations between process variables considered in experiments.

	NETTLE				SAGE			
Correlations	p	t	P	Cp	p	t	P	Cp
p	1	0	0	0.569	1	0	0	0.540
t	0	1	0	0.39	0	1	0	0.412
P	0	0	1	0.44	0	0	1	0.435
Cp	0.569	0.39	0.44	1	0.540	0.412	0.435	1

**Table 7:** Statistical regression polynomial coefficients, rejection probabilities, and coefficient of determination for the dependent variable $C_{p}$.

Term	Coef. Gr. 1	Prob.	Coef. Gr. 2	Prob.
$p^{0}t^{0}P^{0}$	−4.479037	0.0420000	34.194546	0.033431
p	1.166000	0.0001099	−3.886500	0.219219
t	0.026650	0.0042100	−0.275558	0.002799
P	0.045036	0.0015850	−0.166040	0.224380
pt	-	-	0.036453	0.000049
pP	-	-	0.003704	0.366373
tP	-	-	−0.000064	0.385658
$p^{2}$	-	-	0.116778	0.360198
$t^{2}$	-	-	0.000500	0.116437
$P^{2}$	-	-	0.000973	0.155541

**Table 8:** Minimum and maximum values of polyphenol content in nettle plant extract, for multivariate linear regression (2).

Extreme Values	p, Bar	t, Min	P, W	Numeric Cp %	Experimental Cp %
Minimum value	5	60	80	6.553	6.589
Maximum value	7	120	120	12.285	13.713

**Table 9:** Statistical regression polynomial coefficients, rejection probabilities, and coefficient of determination for the dependent variable $C_{p}$.

Term	Coef. Pol. Gr. 1	Prob. of Refusal	Coef. Pol. Gr. 2	Prob. of Refusal
$p^{0}t^{0}P^{0}$	5.394167	0.095927	5.117111	0.387644
p	1.656444	0.000273	−2.469139	0.372563
t	0.042085	0.003452	−0.005731	0.392874
P	0.066636	0.002225	0.382536	0.204218
pt	-	-	0.015967	0.209057
pP	-	-	−0.006154	0.376541
tP	-	-	0.001414	0.061869
$p^{2}$	-	-	0.275333	0.353078
$t^{2}$	-	-	−0.001052	0.118942
$P^{2}$	-	-	−0.002031	0.160615

**Table 10:** Minimum and maximum values of total polyphenol content in sage plant extract, for multivariate linear regression (5).

Extreme Values	p, Bar	t, Min	P, W	Numeric Cp, %	Experimental Cp, %
Minimum value	5	60	80	21.532	20.041
Maximum value	7	120	120	30.036	30.789

## Data Availability

The original contributions presented in the study are included in the article, further inquiries can be directed to the corresponding author.

## References

[1] Ciupei D., Colișar A., Leopold L., Stănilă A., Diaconeasa Z.M. (2024). Polyphenols: From Classification to Therapeutic Potential and Bioavailability. Foods.

[2] Singla R.K., Dubey A.K., Garg A., Sharma R.K., Fiorino M., Ameen S.M., Haddad M.A., Al-Hiary M. (2019). Natural Polyphenols: Chemical Classification, Definition of Classes, Subcategories, and Structures. J. AOAC Int..

[3] De Rossi L., Rocchetti G., Lucini L., Rebecchi A. (2025). Antimicrobial Potential of Polyphenols: Mechanisms of Action and Microbial Responses—A Narrative Review. Antioxidants.

[4] Rao M.J., Zheng B. (2025). The Role of Polyphenols in Abiotic Stress Tolerance and Their Antioxidant Properties to Scavenge Reactive Oxygen Species and Free Radicals. Antioxidants.

[5] Zagoskina N.V., Zubova M.Y., Nechaeva T.L., Kazantseva V.V., Goncharuk E.A., Katanskaya V.M., Baranova E.N., Aksenova M.A. (2023). Polyphenols in Plants: Structure, Biosynthesis, Abiotic Stress Regulation, and Practical Applications (Review). Int. J. Mol. Sci..

[6] Jan R., Khan M., Asaf S., Lubna, Asif S., Kim K.-M. (2022). Bioactivity and Therapeutic Potential of Kaempferol and Quercetin: New Insights for Plant and Human Health. Plants.

[7] Pinto T., Aires A., Cosme F., Bacelar E., Morais M.C., Oliveira I., Ferreira-Cardoso J., Anjos R., Vilela A., Gonçalves B. (2021). Bioactive (Poly)phenols, Volatile Compounds from Vegetables, Medicinal and Aromatic Plants. Foods.

[8] Rudra A., Arvind I., Mehra R. (2021). Polyphenols: Types, sources and therapeutic applications. Int. J. Home Sci..

[9] Prisa D., Menci G. (2024). Innovative fertilizers with added plant extracts in the cultivation of *Valeriana officinalis* and *Raphanus sativus* and in the control of Botrytis and powdery mildew. GSC Biol. Pharm. Sci..

[10] da Silva L.E., Marcatto G.Z., de Souza Gallo A., Forti V.A. (2024). Plants extracts as germination and seedling establishment promoters in lettuce and maize. Ciência Rural.

[11] Soppelsa S., Cellini A., Donati I., Buriani G., Spinelli F., Andreotti C. (2024). Green Alternatives for the Control of Fungal Diseases in Strawberry: In-Field Optimization of the Use of Elicitors, Botanical Extracts and Essential Oils. Horticulturae.

[12] Tăbărașu A.-M., Nenciu F., Anghelache D.-N., Vlădut V.-N., Găgeanu I. (2024). Hybrid Percolation Ultrasound Method for Extracting Bioactive Compounds from *Urtica dioica* and *Salvia officinalis*. Agriculture.

[13] Nenciu F., Fatu V., Arsenoaia V., Persu C., Voicea I., Vladut N.-V., Matache M.G., Gageanu I., Marin E., Biris S.-S. (2023). Bioactive Compounds Extraction Using a Hybrid Ultrasound and High-Pressure Technology for Sustainable Farming Systems. Agriculture.

[14] Repajić M., Cegledi E., Zorić Z., Pedisić S., Elez Garofulić I., Radman S., Palčić I., Dragović-Uzelac V. (2021). Bioactive Compounds in Wild Nettle (*Urtica dioica* L.) Leaves and Stalks: Polyphenols and Pigments upon Seasonal and Habitat Variations. Foods.

[15] Toplicean I.-M., Ianuș R.-D., Datcu A.-D. (2024). An Overview on Nettle Studies, Compounds, Processing and the Relation with Circular Bioeconomy. Plants.

[16] Cenobio-Galindo A.d.J., Hernández-Fuentes A.D., González-Lemus U., Zaldívar-Ortega A.K., González-Montiel L., Madariaga-Navarrete A., Hernández-Soto I. (2024). Biofungicides Based on Plant Extracts: On the Road to Organic Farming. Int. J. Mol. Sci..

[17] Tăbărașu A.-M., Găgeanu I., Anghelache D.-N., Catană L., Vlăduț N.-V., Nenciu F. Evaluation of the biostimulatory effects of nettle and sage extracts on the development of green beans. Proceedings of the 50th Symposium “Actual Tasks on Agricultural Engineering”.

[18] Otles S., Yalcin B. (2012). Phenolic Compounds Analysis of Root, Stalk, and Leaves of Nettle. Sci. World J..

[19] Dent M., Fuchs-Godec R., Pedisić S., Grbin D., Dragović-Uzelac V., Ježek D., Bosiljkov T. (2024). Polyphenols from Sage Leaves (*Salvia officinalis* L.): Environmentally Friendly Extraction under High Hydrostatic Pressure and Application as a Corrosion Inhibitor for Tinplate. Separations.

[20] Mocan A., Babotă M., Pop A., Fizeșan I., Diuzheva A., Locatelli M., Carradori S., Campestre C., Menghini L., Sisea C.R. (2020). Chemical Constituents and Biologic Activities of Sage Species: A Comparison between *Salvia offcinalis* L., *S. glutinosa* L. and S. transsylvanica (Schur ex Griseb. & Schenk) Schur. Antioxidants.

[21] Wafa S., Hanan S.N., Kheira S., Abderrezzak K., Cherif A. (2022). Total phenolic, flavonoid and tannin contents and antioxidant activities of extracts of *Urtica dioica* L. by different extraction techniques. Nat. Resour. Sustain. Dev..

[22] Tarasevičienè Ž., Vitkauskaitè M., Paulauskienè A., Černiauskienè J. (2023). Wild Stinging Nettle (*Urtica dioica* L.) Leaves and Roots Chemical Composition and Phenols Extraction. Plants.

[23] Ðurović S., Micić D., Šorgić S., Popov S., Gašić U., Tosti T., Kostić M., Smyatskaya Y.A., Blagojević S., Zeković Z. (2023). Recovery of Polyphenolic Compounds and Vitamins from the Stinging Nettle Leaves: Thermal and Behavior and Biological Activity of Obtained Extracts. Molecules.

[24] Koraqi H., Qazimi B., Khalid W., Stanoeva J.P., Sehrish A., Siddique F., Çesko C., Khan K.A., Rahim M.A., Hussain I. (2023). Optimized conditions for extraction, quantification and detection of bioactive compound from Nettle (*Urtica dioica* L.) using the deep eutectic solvents, ultra-sonication and liquid chromatography-mass spectrometry (LC-DAD-ESI MS/MS). Int. J. Food Prop..

[25] Ei E., Park H.H., Kuk Y.I. (2025). Growth Promotion and Secondary Metabolites of Vegetables by Spraying Soil with *Psidium guajava*, *Aloe vera*, *Allium sativum* and *Medicago sativa* Extracts at Various Stages of Growth. Plants.

[26] Rouphael Y., Giordano M., Cardarelli M., Cozzolino E., Mori M., Kyriacou M.C., Bonini P., Colla G. (2018). Plant- and Seaweed-Based Extracts Increase Yield but Differentially Modulate Nutritional Quality of Greenhouse Spinach through Biostimulant Action. Agronomy.

[27] Hegazy M.G.A., Ahmed A.-R.M., Yousef A.F., Ali W.M., Nasr A., Elshazly E.H., Shalaby M.E., Teiba I.I., Al-Bedak O.A.M. (2024). Effectiveness of some plant extracts in biocontrol of induced onion basal rot disease in greenhouse conditions. AMB Express.

[28] Greff B., Sáhó A., Lakatos E., Varga L. (2023). Biocontrol Activity of Aromatic and Medicinal Plants and Their Bioactive Components against Soil-Borne Pathogens. Plants.

[29] Salihovic E., Salkic B., Imsirovic E., Hodzic S., Nocajevic S., Salkic A. (2022). Influence of Biopesticides and Natural Preparations on the Regulation of Diseases and Pests in the Ecological Protection of Tomatoes (*Lycopersicon esculentum* L.) and Cucumbers (Cucumis sativus L.). J. Appl. Life Sci. Int..

[30] Yorulmaz Salman S., Saritaș S., Kara N., AY R. (2014). Acaricidal and Ovicidal Effects of Sage (*Salvia officinalis* L.) and Rosemary (*Rosmarinus officinalis* L.) (Lamiaceae) Extracts on *Tetranychus urticae* Koch (Acari: Tetranychidae). J. Agric. Sci..

[31] Jassam Y.A., Kareem T.A. (2019). The effect of plant extracts of sage (*Salvia officinalis*) and thyme (*Thymus vulgaris*) in the cause of disease complicating the roots of nematodes meloidogyne incognita on the tomato in Iraq. Plant Arch..

[32] Proestos C., Komaitis M. (2006). Ultrasonically assisted extraction of phenolic compounds from aromatic plants: Comparison with conventional extraction technics. J. Food Qual..

[33] Dent M., Dragovic-Uzelac V., Elez Garofulic I., Bosiljkov T., Jezek D., Brncic M. (2015). Comparison of Conventional and Ultrasound-assisted Extraction Techniques on Mass Fraction of Phenolic Compounds from Sage (*Salvia officinalis* L.). Chem. Biochem. Eng. Q..

[34] Velicković D.T., Milenović D.M., Ristić M.S., Veljković V.B. (2006). Kinetics of ultrasonic extraction of extractive substances from garden (*Salvia officinalis* L.) and glutinous (*Salvia glutinosa* L.) sage. Ultrason. Sonochemistry.

[35] Šic Žlabur J., Radman S., Opačić N., Rašić A., Dujmović M., Brnčić M., Barba F.J., Castagnini J.M., Voća S. (2022). Application of Ultrasound as Clean Technology for Extraction of Specialized Metabolites from Stinging Nettle (*Urtica dioica* L.). Front. Nutr..

[36] Di Mola I., Cozzolino E., Ottaiano L., Giordano M., Rouphael Y., Colla G., Mori M. (2019). Effect of Vegetal- and Seaweed Extract-Based Biostimulants on Agronomical and Leaf Quality Traits of Plastic Tunnel-Grown Baby Lettuce under Four Regimes of Nitrogen Fertilization. Agronomy.

[37] Chrysargyris A., Charalambous S., Xylia P., Litskas V., Stavrinides M., Tzortzakis N. (2020). Assessing the Biostimulant Effects of a Novel Plant-Based Formulation on Tomato Crop. Sustainability.

[38] Al-Hamdani A., Jayasuriya H., Pathare P.B., Al-Attabi Z. (2022). Drying Characteristics and Quality Analysis of Medicinal Herbs Dried by an Indirect Solar Dryer. Foods.

[39] Poós T., Varju E. (2017). Drying characteristics of medicinal plants. Int. Rev. Appl. Sci. Eng..

[40] Horszwald A., Andlauer W. (2011). Characterisation of bioactive compounds in berry juices by traditional photometric and modern microplate methods. J. Berry Res..

[41] Ryazi E., Rakhshandehroo F., Abdossi V. (2022). The effect of the aquatic and alcoholic extracts of bipod nettle weed on the growth and some biochemical features of tomato plant under the greenhouse condition. Q. Sci. J. Appl. Biol..

[42] Maričić B., Radman S., Romić M., Perković J., Major N., Urlić B., Palčić I., Ban D., Zorić Z., Ban S.G. (2021). Stinging Nettle (*Urtica dioica* L.) as an Aqueous Plant-Based Extract Fertilizer in Green Bean (*Phaseolus vulgaris* L.) Sustainable Agriculture. Sustainability.

[43] Maričić B., Brkljača M., Ban D., Palčić I., Franin K., Marcelić Š., Goreta Ban S. (2022). Non-Aerated CommonNettle (*Urtica dioica* L.) Extract Enhances Green Beans (*Phaseolus vulgaris* L.) Growth and Soil Enzyme Activity. Life.

[44] Waked D.A. (2016). Bio-efficacy assessment of sage, *Salvia officinalis* L. Extracts on some biological aspects of spider mite, *Tetranychus urticae* koch (Acari: Tetranychidae). Egypt. J. Agric. Res..

[45] Knezevic B., Boskovic N., Zoric M., Leka Z.B. Macro and microelements in the leaf and extract of nettle from different localities of Montenegro. Proceedings of the 2nd International Conference on Chemo and Bioinformatics.

[46] Then M., Vasarhelyi-Peredi K., Szollosy R., Szentmihalyi K. (2004). Polyphenol-, Mineral Element Content and Total Antioxidant Power of Sage (*Salvia officinalis* L.) Extracts. Acta Hort..

[47] Peterson R., Jensen P. (1986). Effects of Nettle Water on Growth and Mineral Nutrition of Plants. II. Pot- and Water-Culture Experiments. Biol. Agric. Hortic..

[48] Statistics Kingdom Multiple Linear Regression Calculator. https://www.statskingdom.com/410multi_linear_regression.html.

[49] PTC Statistics of Multivariate Polynomial Regression. https://support.ptc.com/help/mathcad/r9.0/en/index.html#page/PTC_Mathcad_Help/statistics_of_multivariate_polynomial_regression.html.

